# A CARE-compliant article: A case report and literature review of IgG4-related lung disease

**DOI:** 10.1097/MD.0000000000032075

**Published:** 2022-11-25

**Authors:** Hongmei Ren, Zhiqing Han, Xiaoming Zuo, Jinping Wang, Siming Meng, Cuixia Zheng

**Affiliations:** a Department of Respiratory Critical Medicine, Yangpu Hospital of Tongji University, Shanghai, China; b Department of Pathology, Yangpu Hospital of Tongji University, Shanghai, China.

**Keywords:** IgG4-related diseases, lung

## Abstract

IgG4-related lung disease (IgG4-RLD) is an unusual disease, with various clinical manifestations and various chest imaging findings. The patients may have no respiratory symptoms. Therefore, diagnosis is challenging. This can easily cause misdiagnosis and mistreatment.

**Patient concerns:**

A 71-year-old male presented with chest pain, cough, and shortness of breath. Plain chest computed tomography scans showed multi-locus nodes at the center of the hilum.

**Diagnosis:**

Percutaneous lung biopsy was performed, and IgG4-RLD was diagnosed.

**Interventions:**

Prednisone was orally administered daily.

**Outcomes:**

The case’s symptoms improved. The patient was discharged from the hospital. After 2 months of reexamination, his symptoms were relieved. Reexamination of the chest computed tomography showed that multi-locus nodes of the lung were obviously absorbed compared with those before.

**Lessons:**

IgG4-RLD is a rare respiratory disease. It has atypical clinical manifestations and chest images. We report the first case of IgG4-RLD showing multi-locus nodes centered on the hilar, hypertrophic mucosa; as well as a narrow and even occluded lumen.

## 1. Introduction

IgG4-related disease (IgG4-RD) is a newly recognized autoimmune-mediated fibroinflammatory disease that may affect multiple organs and lead to tumefactive, tissue-destructive lesions and organ failure. It is characterized by a dense lymphoplasmacytic infiltrate rich in IgG4-positive plasma cells, storiform fibrosis, and often but not always, elevated serum IgG4 concentrations.^[1,2]^ The involvement of nearly every anatomical site has been previously reported. The most commonly involved organs are the pancreas, bile duct, main salivary gland (submandibular gland and parotid gland), lacrimal gland, retroperitoneum and lymph nodes.^[3,4]^ Immunoglobulin G4 related disease was first reported in the late 1990s. Yoshida et al^[5]^ reported that a large number of IgG4 positive plasma cells infiltrated in a patient with autoimmune pancreas (AIP). In 2003, it was reported for the first time that a large number of IgG4-positive plasma cells infiltrated the lung lesions of patients with AIP. It has been suggested that AIP is a new autoimmune disease related to IgG4 with multiple system involvement, which may lead to lung involvement. In 2009, Zen et al^[6]^ called IgG4-RD involved in lung “IgG4-related lung disease (IgG4-RLD).” Owing to the late recognition of IgG4-RLD and lack of clinical experience, it is easy to cause misdiagnosis and mistreatment. Here, we report a pathologically confirmed case of IgG4-RLD with multi-locus nodes were centered on the hilum, and review the relevant literature to improve the understanding of the disease.

## 2. Case presentation

**Figure 1.**
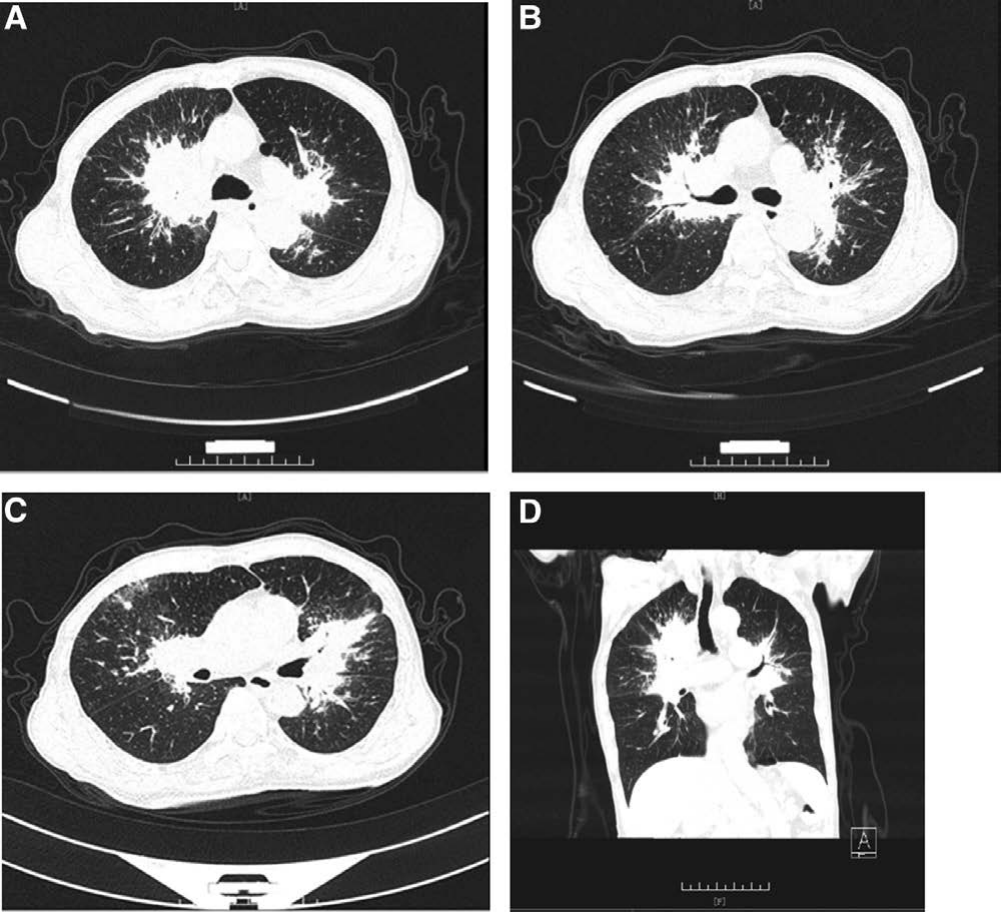
(A–D) 2020-03-19 Chest CT showed multi-locus nodes in the upper right, middle right, upper left and lower left lobes. These shadows were centered on the hilum, with an unclear boundary. Local bronchial lumen stenosis, miliary nodular shadows in both lungs, and an enlarged hilar area were also observed. CT = computed tomography.

**Figure 2.**
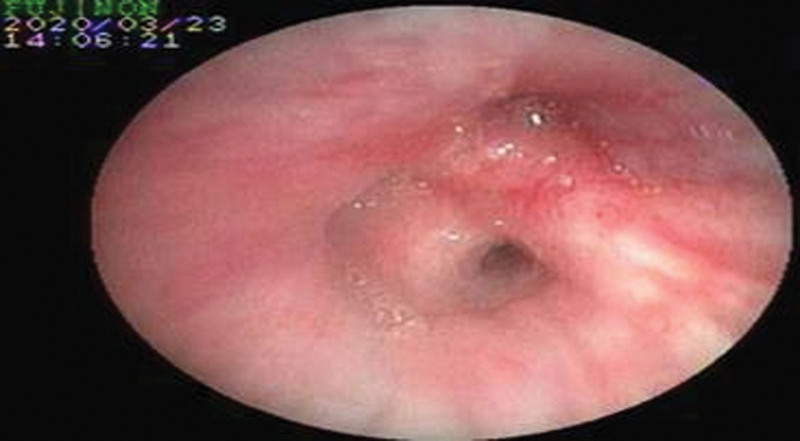
2020-03-23 Bronchoscopy showed that mucosa of the upper left, upper right, middle right, and lower left lobes was hypertrophic. The lumen was narrow and the apical segment of the upper right lobe was occluded.

**Figure 3.**
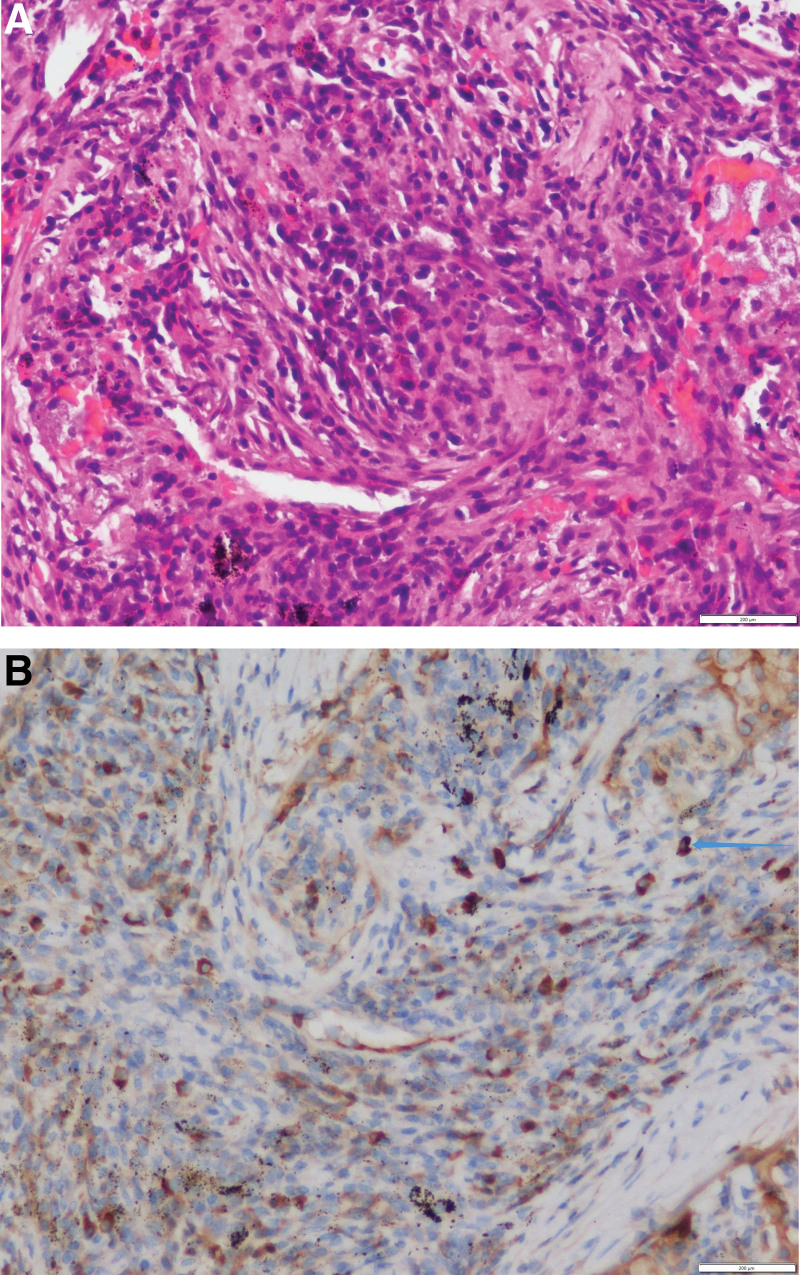
(A) Lung biopsy showed nodular and storiform fibrosis and hyperplasia in the interstitial tissue of the lung. In addition, dense lymphoplasmacytic infiltration was observed. HE 400 times. (B) The results of lung biopsy showed that the number of IgG4 positive plasma cells (arrows) was >10/HPF. HE 400 times. HPF = high power field.

**Figure 4.**
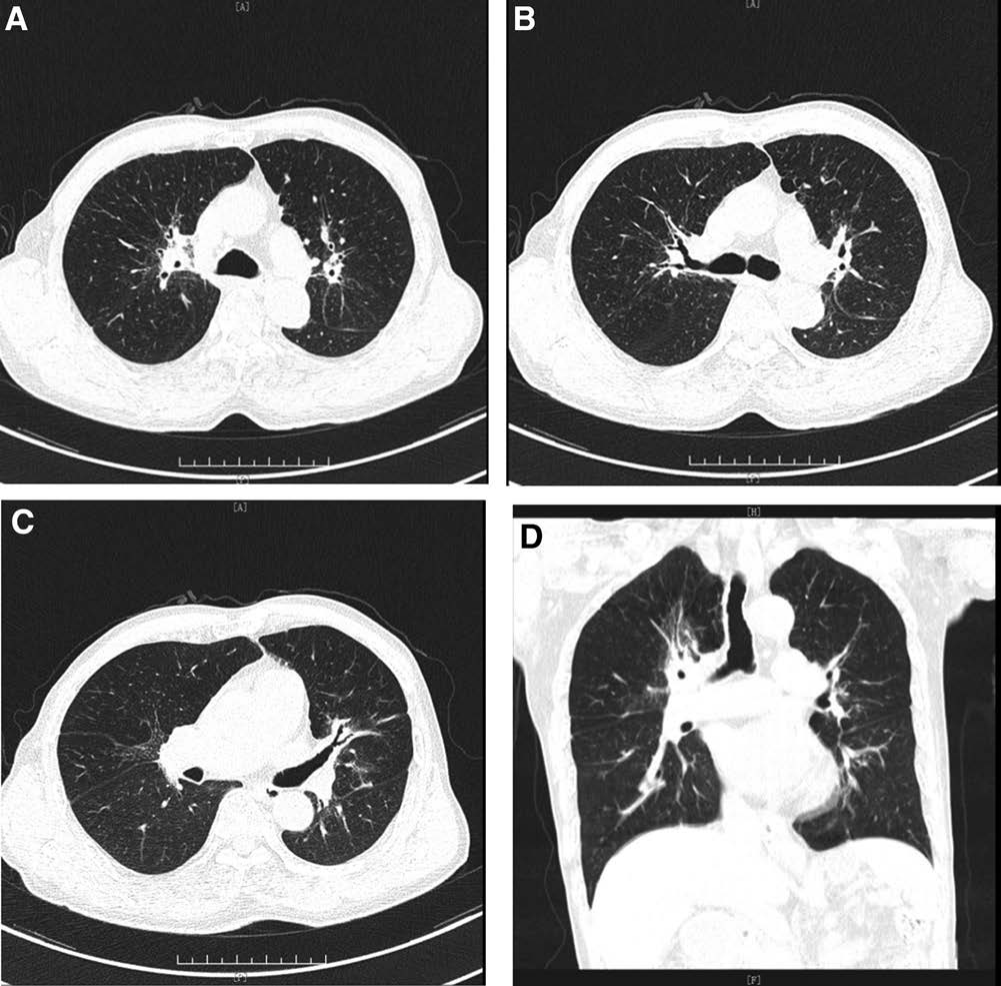
(A–D) 2020-06-04 Bilateral multi-locus nodes were absorbed obviously.

A 71-year-old male presented with chest pain, cough, and shortness of breath for 1 month, with aggravated symptoms for 1 week. One month before admission, the patient fell and complained of chest pain, shortness of breath, and cough with a small amount of white phlegm. The chest pain was paroxysmal dull pain in the lower chest, which was heavier on the right side than on the left side and had no correlation with the change in breath and posture. With a progressive decrease in poor appetite and fatigue, he visited the respiratory clinic of our hospital. A plain chest computed tomography (CT) scan showed multi-locus nodes in the upper right, middle right, upper left, and lower left lobes. These shadows were centered on the hilum, with an unclear boundary. Local bronchial lumen stenosis, miliary nodular shadows in both lungs, and an enlarged hilar area were also observed (Fig. 1). He had a history of lacunar cerebral infarction for 10 months and was taking long-term oral aspirin. His respiratory rate was 22 breaths/min, and he experienced slight shortness of breath. In addition, there were no positive signs in the lung and no obvious abnormalities in the rest. Auxiliary examination demonstrated 72.61 mg/L of serum C-reactive protein (CRP), 115 g/L of hemoglobin (Hb), 59 mm/h of erythrocyte sedimentation rate (ESR), 31.9 g/L of serum albumin, 3.05 mmol/L of serum potassium, and 0.05 Ng/mL of serum procalcitonin. His urine and fecal routine, electrolytes of the liver and kidney function, fungal D-glucan, and thyroid function were normal. Blood tumor markers, including carcinoembryonic antigen, sugar antigen CAl9-9, squamous cell carcinoma antigen, neuron-specific enolase, and cytokeratin-19-fragment, were normal. His antinuclear antibody and antineutrophil cytoplasmic antibody results were negative, and his creatine kinase level was normal. Rheumatoid factor was 110 IU/mL (reference value <15 IU/mL); Immunoglobulin G was 14.3 g/L (reference value 7–16 g/L); Immunoglobulin G4 was 3.4 g/L (reference value 0.03–2.01 g/L); Immunoglobulin A was 2.13 g/L (reference value 0.7–4 g/L); Immunoglobulin M was 1.05 g/L (reference value 0.4–2.3 g/L). The total complement was 55.0 u/mL (reference value 23–46 U/mL). Arterial blood gas analysis showed: a pH of 7.45, 5.18 kpa of PaCO_2_ and 9.2 kpa of PaO_2_ (without oxygen inhalation). No obvious abnormalities were found on an enhanced CT scan of the upper abdomen. Further electron bronchoscopy showed that the bronchial mucosa of the upper left, upper right, middle right, and lower left lobes was hypertrophic. The lumen was narrow and the apical segment of the upper right lobe was occluded (Fig. 2). Pathological examination of the bronchial mucosa on the apical segment of the upper right lobe revealed chronic mucosal inflammation. Percutaneous lung biopsy of the right upper lung was performed. Histopathological examination revealed nodular and storiform fibrosis and hyperplasia in the interstitial tissue of the lung. In addition, dense lymphoplasmacytic infiltration was observed. The number of IgG4- positive plasma cells was >10 per high power field (Fig. 3). According to the diagnostic criteria for IgG4-related respiratory system involvement proposed by the Japanese Respiratory Society in the 54th IgG4-RLD Symposium in 2014,^[7]^ the diagnosis of IgG4-RLD was clear. Thirty milligram of prednisone was orally administered daily, and the symptoms improved. The patient, was discharged from the hospital. After 1 month, the dosage was 25 mg QD administered orally. After 2 months of reexamination, the patient’s symptoms were relieved. The reexamination of chest CT showed that the multi-locus nodes of the lung were obviously absorbed (Fig. 4). The IgG4 level in the serum was 1.93 g/L, which was reduced to normal. At present, the condition is stable, and the follow-up treatment is continuing.

## 3. Discussion

IgG4-RLD is a rare condition. We searched PubMed, CNKI, and Wanfang databasea with the options of All Field “IgG4 related disease” AND All Field“lung,” AND All Field “IgG4 related disease” AND All Field “respiratory.” From 1990 to May 2020, 75 complete lgG4-related lung disease studies, 56 in English and 19 in Chinese, were retrieved. All of these studies were confirmed by histopathology, with a total of 189 cases. Most were case reports, 17 of which involved >2 cases, including one that involved 21 cases (Table 1).

**Table 1:** General clinical data of IgG4-related lung disease (IgG4-RLD) reviewed.

Researcher	Number of cases	Average age (yr)	Gender (male/female)	Number of cases with respiratory symptoms
Yoh Zen et al 2009	21	69	17/4	10, where: Cough (10) Hemoptysis (10)
Shoko Matsui et al 2013	18	62	14/4	5
Xuefeng sun et al 2016	17	44.8	6/11	Cough (11) dyspnea (5) chest pain (4)
Dai Inoue et al 2012	13	62	9/4	10, where: Cough (7) hemoptysis (2) exertional dyspnea (2) chest pain (1)
Ming Wu et al 2018	13	51.1	8/5	10, where: Cough (9) expectoration (6) hemoptysis (6) chest distress (2) asthma (2)
Guojing Han et al 2017	8	59	4/4	2, where: Shortness of breath (2), cough (2), expectoration (2) after exercise
Xiaoting LV et al 2018	7	56	4/3	6, where: Dyspnea (6) cough (2)
Yan Li et al 2018	6	55	5/1	6, where: Cough (5) expectoration (4) asthma (3) shortness of breath after activity (1) chest pain (1) chest distress (1) hemoptysis (1)
Joseph C Keenan et al 2016	6	55.2	5/1	4, where: Cough (2) hemoptysis (1) dyspnea (1)
HuiZhang et al 2012	4	47.8	1/3	4, where: Dry cough (3) shortness of breath (3) chest tightness (1)
Phillip Hui et al 2013	3	52	1/2	3, where: Cough (3) dyspnea (2) expectoration (2)
Erik baltaxe et al 2016	3	41	2/1	2, where: Cough (1) dyspnea (1) expectoration (1) pleural pain (1)
Kyoko Yamashita et al 2008	3	72	3/0	Exertive dyspnea (2)
Xuefeng sun, min Peng et al 2014	3	42.5	1/2	3, where: Cough (3) expectoration (1) intermittent hemoptysis (1) chest pain (1)
MingxiaRen et al 2019	2	67	2/0	Cough and expectoration (1)
Masahiro Kitada et al 2015	2	61.5	1/1	Phlegm Blood (1)
XinghuaShan et al 2015	2	45.5	2/0	Cough (2) expectoration (2) Phlegm Blood (1) chest pain (1) chest distress (1)
Case report	58	60	44/15	45, where: Cough (21) expectoration (9) dyspnea (7) dry cough (7) laborious dyspnea (6) shortness of breath after activity (5) Phlegm Blood (4) hemoptysis (2) asthma (2) chest pain (3) back pain (1)

The 75 papers reviewed in this study were published between 2007 and 2020. The authors of these papers include researchers from China, Japan, the United States, South Korea, the United Kingdom, Germany, Italy, Australia, Greece, Argentina and other countries. Most of the papers published were from Japan, with scientists contributing 25 papers. However, the prevalence and incidence of IgG4-RD remain unclear. According to Japanese scholars, the incidence of this disease throughout Japan is 0.28 to 1.08/100,000.^[8]^ 14 to 35% of patients with IgG4-RD will have intrathoracic invasion.^[9,10]^ IgG4-RD is mainly found in adults, most of them are men. Of the 189 cases, 133 were male and 56 were female, aged 23 to 79 years old, with an average age of 55.7 years old (Table 1).

The clinical manifestations of IgG4-RLD were various. The literature review shows that among 126 of 189 patients with respiratory symptoms, such as cough, expectoration, dyspnea, chest pain, hemoptysis, and shortness of breath after activity, 24 patients initially showed extrapulmonary symptoms, and 13 patients with asymptomatic physical examination were confirmed by chest imaging and pathology of the lung (Table 1). In terms of clinical manifestations, these symptoms lack specificity and are difficult to distinguish from those of other respiratory diseases. Moreover, they are prone to misdiagnosis and missed diagnose.

An increase lgG4 concentration in serum is an important feature of laboratory examinations. Nevertheless, the increase in serum IgG4 levels was not specific. It can be seen in 5% of normal people or in 10% of patients with malignant tumors of the biliopancreatic system or other infectious diseases and inflammatory diseases.^[11]^ The sensitivity and specificity of the diagnosis of IgG4-RD through serum lgG4 levels were 63% and 94%, respectively.^[12,13]^ 3 to 30% of patients had normal serum IgG4 level.^[2]^

The chest imaging manifestations of IgG4-RLD vary with more than half of the cases reviewed showing nodule/mass shadows in 105 cases (55.5%). Five cases of chest imaging showed cavities, 2 cases showed cystic lesions, and 1 indicated emphysema. Imaging manifestations can occur alone or in combination with several types of lesions. The above situation shows that although the imaging manifestations of IgG4-RLD have certain characteristics, they lacks obvious specificity. Nodular shadows and mass shadows should be differentiated from lung cancer. Interstitial changes in the alveoli, enlargement of hilar/mediastinal lymph nodes, and thickening of the bronchial wall and vascular bundle should be differentiated from diffuse lung diseases such as lymphoproliferative diseases, sarcoidosis and nonspecific interstitial pneumonia.

Histopathological examination is the key to the diagnosis of the disease, and tissue biopsy is important to exclude malignant diseases, tuberculosis, interstitial pneumonia, organic pneumonia, Castleman disease, and other diseases. A total of 189 patients had definite histopathology. Pathological specimens from 161 patients were obtained by pulmonary resection, video-assisted thoracic surgery, lung biopsy, percutaneous lung biopsy, and bronchoscopy lung/mucosa biopsy. Two cases were diagnosed by pleural biopsy, and 19 by extrapulmonary organ biopsy. Although there were no lung tissue biopsy data, the lung images decreased or even disappeared following treatment; thus, IgG4-RLD was confirmed. Histopathological features: in 184 biopsies, lymphocytic plasmacytosis was observed in 132 biopsies, IgG4 positive plasmacytosis was found in 10 to 159 (number/ HP). The IgG4/IgG plasma cell ratio in 156 tissues was between 23% and 88%. There were obliterative phlebitis in 49 tissues, obliterative arteritis in 23 tissues, storiformfibrosis in 82 tissues and irregular fibrosis in 38 tissues. In addition, a small number of IgG4 positive cells can also be seen in many inflammatory infiltrates; therefore, in absolute quantity, the number of IgG4 positive plasma cells determined in the literature is between 10 and 50/high power field. If the ratio of IgG4 positive plasma cells to IgG plasma cells is >50%, it would be more helpful in diagnosing IgG4-RD. In particular, in the late stage of the disease, when only a small number of plasma cells exist in the tissue and fibrosis becomes the main component, the ratio of IgG4 to IgG plays a key role in the diagnosis.^[14]^

IgG4-RLD responded well to glucocorticoid therapy. More than 50% of the 189 patients reviewed in this study were treated with glucocorticoids alone, with an effective rate of 87.6%. Commonly used glucocorticoids include prednisone, prednisolone or methylprednisolone, with an initial dosage of 0.5 to 1 mg/kg/day for prednisone, 0.5 to 0.6 mg/kg/day for prednisolone or 0.4 to 80 mg/kg/day for methylprednisolone. A total of 9.3% of patients experienced recurrenced after glucocorticoid reduction, and the symptoms improved after glucocorticoid addition or combination therapy with immunosuppressive agents. The immunosuppressive agents used in this review included methotrexate, mycophenolate mofetil, azathioprine, cyclophosphamide, and cyclosporine. The effective surgical rate was 56%. Fourteen cases were untreated, and 2 cases were self-healing (self-healing rate was 14.3%), indicating that the disease had some self-healing. In particular, fluticasone propionate (500 μg) inhaled with salmeterol (50 μg) was administered twice a day. Two cases of glucocorticoids combined with rituximab have been reported in the literature. Although they were effective, the disease relapsed easily. When the drugs were reduced, the disease recurred easily. Currently, the optimal maintenance dose and time for IgG4-RLD have not been determined. After treatment, patients who stop the drug and relapse can be treated with glucocorticoids again. Immunosuppressive agents should be used to maintain remission. However, the prognosis of this disease remains unclear. Of the 189 patients reviewed in this study, only 3 died and 1 died after video-assisted thoracic surgery.

In summary, IgG4-RLD is a rare respiratory disorder. It has atypical clinical manifestations and chest images. It can exist alone and is often associated with other systemic diseases. Moreover, it is difficult to distinguish it from common respiratory diseases, such as lung cancer, tuberculosis, and the diagnosis of the disease depends on clinical, chest images, serum IgG4 levels, and comprehensive pathological analysis. The patient responded well to glucocorticoid treatment. Some cases are prone to recurrence in the process of hormone reduction; therefore, they should be followed up for a long time after treatment. However, its prognosis is unclear. Therefore, further prospective multicenter clinical studies are required.

## 4. Conclusions

Our case implied 3 clinical evidences: first, multi-locus nodes 1n the middle of the hilar could be the presentation of IgG4-related respiratory disease. The second is, IgG4-related respiratory disease with hypertrophic mucosa. Finally, IgG4-related lung disease can be very sensitive to prednisone. Lung lesions were absorbed further, the serum concentration of IgG4 decreased after prednisone treatment, and the symptoms significantly improved in our case. In brief, we report the first case of IgG4-RLD showing multi-locus nodes centered on the hilar, hypertrophic mucosa, as well as a narrow and even occluded lumen. These findings further our understanding of the characteristics of IgG4-RLD.

## Author contributions

HR contributed to the conception and design, all data collection, and analysis and interpretation of data and played a major role in the writing of the article. ZH, JW and SM provided raw data of the patient. XZ provided suggestions for documenting the pathological findings in this report, and CZ made key revisions to the manuscript for important intellectual content. All authors have read and provided consent for the final manuscript, and have ensured the integrity of their respective intellectual parts.

**Conceptualization:** Hongmei Ren.

**Data curation:** Hongmei Ren.

**Formal analysis:** Hongmei Ren.

**Funding acquisition:** Hongmei Ren.

**Investigation:** Hongmei Ren, Jinping Wang, Siming Meng.

**Methodology:** Hongmei Ren.

**Project administration:** Hongmei Ren.

**Resources:** Hongmei Ren, Zhiqing Han, Xiaoming Zuo, Jinping Wang, Siming Meng.

**Supervision:** Hongmei Ren, Cuixia Zheng.

**Validation:** Hongmei Ren.

**Writing – original draft:** Hongmei Ren.

**Writing – review & editing:** Hongmei Ren, Cuixia Zheng.
